# Point-of-care Ultrasound in Early Diagnosis of Cardiomyopathy in a Child with Viral Myocarditis: A Case Report

**DOI:** 10.5811/cpcem.2021.2.51266

**Published:** 2021-04-19

**Authors:** Ayush Gupta, Trevor Eckenswiller

**Affiliations:** * Children’s Hospital of New Orleans, Department of Pediatric Emergency Medicine, New Orleans, Louisiana; † William Beaumont Hospital, Department of Emergency Medicine, Royal Oak, Michigan

**Keywords:** POCUS, myocarditis, diagnostics, case report, pediatric

## Abstract

**Introduction:**

Pediatric myocarditis is a commonly missed diagnosis in the pediatric emergency department (ED) with high morbidity and mortality. The presentation of cardiogenic shock secondary to myocarditis and septic shock can be difficult to differentiate during initial resuscitation, and incorrect treatment can lead to poor prognosis. Early diagnosis may provide a better prognosis for this life-threatening condition.

**Case Report:**

We report a case of a five-year-old female who presented to the ED with non-specific symptoms of myocarditis. Rapid point-of-care ultrasound led to early diagnosis, correct management, and great prognosis for the patient.

**Conclusion:**

Providers must maintain a high index of suspicion for cardiogenic shock in patients with nonspecific symptoms and fluid unresponsiveness. Point-of-care ultrasound can help in the identification of cardiac disorders and guide practitioners in their management plans.

## INTRODUCTION

Point-of-care ultrasonography (POCUS) is a safe, effective imaging modality with a rapidly expanding array of life-saving, time-saving, and cost-saving applications in pediatric emergency medicine. The American Academy of Pediatrics recently published the first policy statement endorsing the use of POCUS by pediatric emergency physicians (EP). An accompanying technical report concluded: “It is our responsibility to our patients to stay abreast of the most current advances in medicine and provide the safest, most efficient, state-of-art care. Point-of-care [ultrasound] will help us meet that goal.” Rapid ultrasound for shock and hypotension (RUSH) exam includes standardized views of the heart, inferior vena cava (IVC), lungs, and abdomen to further categorize the type of shock (eg, hypovolemic, cardiogenic, obstructive, distributive).^1^ Focused cardiac ultrasound enables pediatric EPs trained in its use to diagnose pericardial effusions, assess cardiac contractility, and left ventricular enlargement with 91% accuracy.^2^ It should be considered for assessing patients with signs or symptoms potentially related to cardiac dysfunction or effusion, such as shortness of breath, chest pain, syncope, hypotension/shock, or a new murmur.^3^

Myocarditis is rare in children, with an estimated annual incidence of 1–2 per 100,000 children.^4^ A higher incidence of myocarditis is noted in autopsy studies of infants and children who died suddenly and unexpectedly; evidence of myocarditis was noted in approximately 10–20% of such cases. These data represent that the true incidence of pediatric myocarditis is probably underestimated. The diagnosis is challenging due to the nonspecific symptoms such as respiratory distress or gastrointestinal symptoms (anorexia, abdominal pain, and vomiting), which may be the most prominent features at presentation. Reported mortality rates during the acute illness for children with myocarditis range from 7–15%.^5^

In the pediatric ED several tests aid in the diagnosis of this life-threatening condition. Electrocardiogram (ECG) and troponin levels are performed when the suspicion is high. The RUSH exam conducted to evaluate shock may be of determinant help in assessing cardiac contractility and enlargement. We report a case of a five-year-old female who presented to the pediatric ED with non-specific symptoms of myocarditis. Rapid POCUS led to early diagnosis, correct management, and great outcome for this patient.

## CASE REPORT

A five-year-old female presented to the pediatric ED with a chief complaint of vomiting. According to her parents, the patient had been having three days of fever and vomiting up to 10 times a day, which was non-bilious and non-bloody. She was also noted to have a sore throat, poor appetite, decreased sleep, and mild cough. The patient had seen her primary care physician the day before the pediatric ED visit; rapid streptococcal antigen test was negative, and she was sent home. The patient had no other past medical or surgical problems, no allergies, and was not taking any medications.

Her vitals on presentation was a temperature of 96.6°F orally, heart rate of 159 beats per minute, respiratory rate of 66 breaths per minute, blood pressure of 93/42 millimeters mercury (mm Hg), and oxygen saturation of 98% on room air. The patient was lethargic on examination, had dry mucous membranes, weak peripheral pulses, and capillary refill of 3–4 seconds. She had no abnormal lung sounds or heart murmur, and no hepatosplenomegaly.

In the pediatric ED, she was immediately connected to a monitor, and point-of-care blood glucose was obtained (146 milligrams per deciliter [mg/dL]; normal range 90–130 mg/dL). The differential diagnosis was broad and included dehydration, viral gastroenteritis, sepsis, appendicitis, obstruction, and pneumonia. Due to poor perfusion, no blood tests could be obtained but a peripheral intravenous access was established through which a 20 milliliters per kilogram (kg) normal saline bolus was given rapidly. Her initial capillary blood gas showed a pH of 7.30 (7.35–7.45), partial pressure of carbon dioxide of 26 mm Hg (35–45 mm Hg), and bicarbonate of 12 milliequivalents per liter (mEq/L) (22–28 mEq/L).

A POCUS was conducted (Videos 1, 2, 3) using a Sonosite 5-1 megahertz phased array probe (FUJIFILM Sonosite, Inc., Bothell, WA) to examine the cardiac function. The parasternal long-axis view demonstrated global hypokinesis with left ventricular ejection fraction (LVEF) less than 30%. The right ventricle was mildly dilated. The apical 4-chamber view showed slight pericardial effusion and decreased LVEF. We then conducted the sub-xyphoid IVC view, which showed a plethoric IVC with small pericardial effusion and small pleural effusion posteriorly with B lines.

**CPC-EM Capsule**

What do we already know about this clinical entity?

*Cardiogenic shock is not uncommon in pediatric population but the diagnosis is often missed or delayed due to lack of specific clinic features.*

What makes this presentation of disease reportable?

*This child presented with non-specific symptoms of myocarditis but the early diagnosis was critical to prevent poor outcome.*

What is the major learning point?

*Point-of-care ultrasound (POCUS) can be useful in diagnosing cardiogenic shock in children.*

How might this improve emergency medicine practice?

*By using POCUS, providers can make early diagnosis and improve outcome of children in the emergency department.*

A chest radiograph (Figure 1) and ECG were performed (Figure 2). The patient was diagnosed with myocarditis with cardiogenic shock based on physical examination and rapid POCUS. The cardiology team agreed with the diagnosis, and troponin later resulted at 30.40 nanograms/mL (normal is <0.03 ng/ml). In the pediatric ED, the patient had worsening mental status, increased tachypnea, and lethargy. Fluids were stopped and epinephrine drip was started, which resulted in improvement in her condition. She was transferred to the pediatric cardiac intensive care unit (PCICU).

The same day in the PCICU, the patient became bradycardic and lost pulses. Cardiopulmonary resuscitation was initiated, and the patient was placed on extracorporeal membrane oxygenation. She stayed in the hospital for 10 days and recovered almost completely. She was discharged with some left-sided weakness, and mild speech and swallowing difficulty for which she is undergoing rehabilitation. The final diagnosis was fulminant viral myocarditis (due to enterovirus) with cardiogenic shock.

## DISCUSSION

Pediatric myocarditis is a commonly missed diagnosis in the pediatric ED. In up to 50% of cases, an etiology is never identified; therefore, most cases are deemed idiopathic. The most common identified source in the developed world is viral. However, it can be caused by systemic disorders, bacteria, fungi, parasites, and toxins. Chagas disease is the most prevalent cause worldwide.^6^ The presentation can vary, but common symptoms can include fatigue, fever, chest discomfort, dyspnea, and vomiting. On examination, tachycardia is usually exaggerated compared to fever or discomfort, but unfortunately only presents 46–58% of the time.^7^ Hepatomegaly in the presence of non-specific symptoms should increase the suspicion.

Electrocardiogram is abnormal in most cases, but not specific. Changes can include sinus tachycardia, widening of QRS complex, and possible ST-segment changes. Pericarditis can be concomitant. Troponin is frequently elevated, and elevated inflammatory markers can aid in diagnosis. The gold standard for diagnosis is cardiac biopsy, which is rarely preformed before the death of the patient.^8^ Signs and symptoms of myocarditis overlap with many other more common diseases. As in our case, the presentation of cardiogenic shock can be difficult to differentiate from hypovolemic or distributive shock during initial resuscitation. Fluid resuscitation needs to be evaluated as volume overload is deleterious, but these patients can present hypovolemic. In such a case, small crystalloid boluses of 5–10 cubic centimeters/kg can be administered slowly. It is to be noted that cardiogenic shock is a form of cold shock and has a low cardiac output and high systemic vascular resistance. Vasopressor selection is often debated, but inotropic support with epinephrine is a reasonable first choice.^9^

Pediatric POCUS is an emerging modality that physicians are becoming more comfortable with implementing in their practice. Visualization with ultrasound is a fast and easy tool that can be used upon arrival before any laboratory tests have been released. The advantages of POCUS also entail immediate diagnosis, increased accuracy, and minimal radiation dose.^10^ In myocarditis with cardiogenic shock, POCUS may reveal global left ventricular or biventricular dysfunction, dilated cardiomyopathy, and reduced LVEF usually from global hypokinesis. Studies have shown conflicting evidence on use of IVC measurements to asses volume status and fluid responsiveness. In general the two poles of volume status, hypovolemia and fluid overload, can be evaluated in the adult patient using IVC measurements. This can be extrapolated to the pediatric population within reason.^11^,^12^,^13^

In this case, the patient was presumed to have septic shock and received rapid fluid bolus until POCUS altered our management. The echocardiogram also allowed effective management of laboratory studies, as it was extremely difficult to obtain blood secondary to poor perfusion. It was elected to send troponin as the only initial serum test until central venous access could be obtained. Implementing POCUS in critically ill-appearing patients with non-specific symptoms can increase the sensitivity and efficiency of detecting myocarditis early in the ED as well as guiding the resuscitation team accordingly.

## CONCLUSION

Providers must maintain a high index of suspicion for cardiogenic shock in patients with nonspecific symptoms and unresponsive to fluids. Myocarditis is the leading cause of cardiogenic shock in the pediatric population. Point-of-care ultrasound can help in the identification of cardiac disorders and guide management in such patients.

## Supplementary Information

Video 1 Parasternal long-axis view. Point-of-care ultrasound in parasternal long-axis view demonstrating global hypokinesis. Left ventricular ejection fraction less than 30%. Right ventricle mildly dilated.
*LV,* left ventricle; *RV,* right ventricle; *LA,* left atrium.

Video 2 Apical 4-chamber view. Point-of-care ultrasound in apical 4-chamber view demonstrating slight pericardial effusion and decreased left ventricular ejection fraction.

Video 3 Sub-xyphoid inferior vena cava (IVC) view. Point-of-care ultrasound in sub-xiphoid IVC view demonstrating a plethoric IVC and a small pericardial effusion. Also noted is a small pericardial effusion posteriorly with B-lines.

## Figures and Tables

**Image 1:**
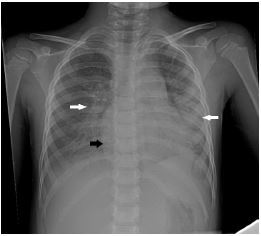
Chest radiograph showing cardiomegaly (black arrow) and pulmonary edema (white arrows).

**Image 2:**
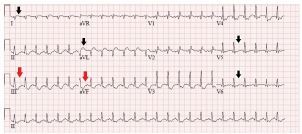
Electrocardiogram showing sinus tachycardia, ST-segment elevation in lateral leads (I, aVL, V5–V6) (black arrows), T-wave inversion in inferior leads (III, aVF) (red arrows).
